# Supplementary material for: TLCD1 and TLCD2 regulate cellular phosphatidylethanolamine composition and promote the progression of non-alcoholic steatohepatitis
Source: Nat Commun. 2022 Oct 14;13:6020. doi: 10.1038/s41467-022-33735-6 (PMC9568529; doi:10.1038/s41467-022-33735-6)
Supplement: Supplementary file 1 — Supplementary information [file 41467_2022_33735_MOESM1_ESM.docx]

**Supplementary Information**

**TLCD1 and TLCD2 regulate cellular phosphatidylethanolamine composition and promote the progression of non-alcoholic steatohepatitis**

**Contains:**

Supplementary Figures 1-11 and Supplementary Figure legends


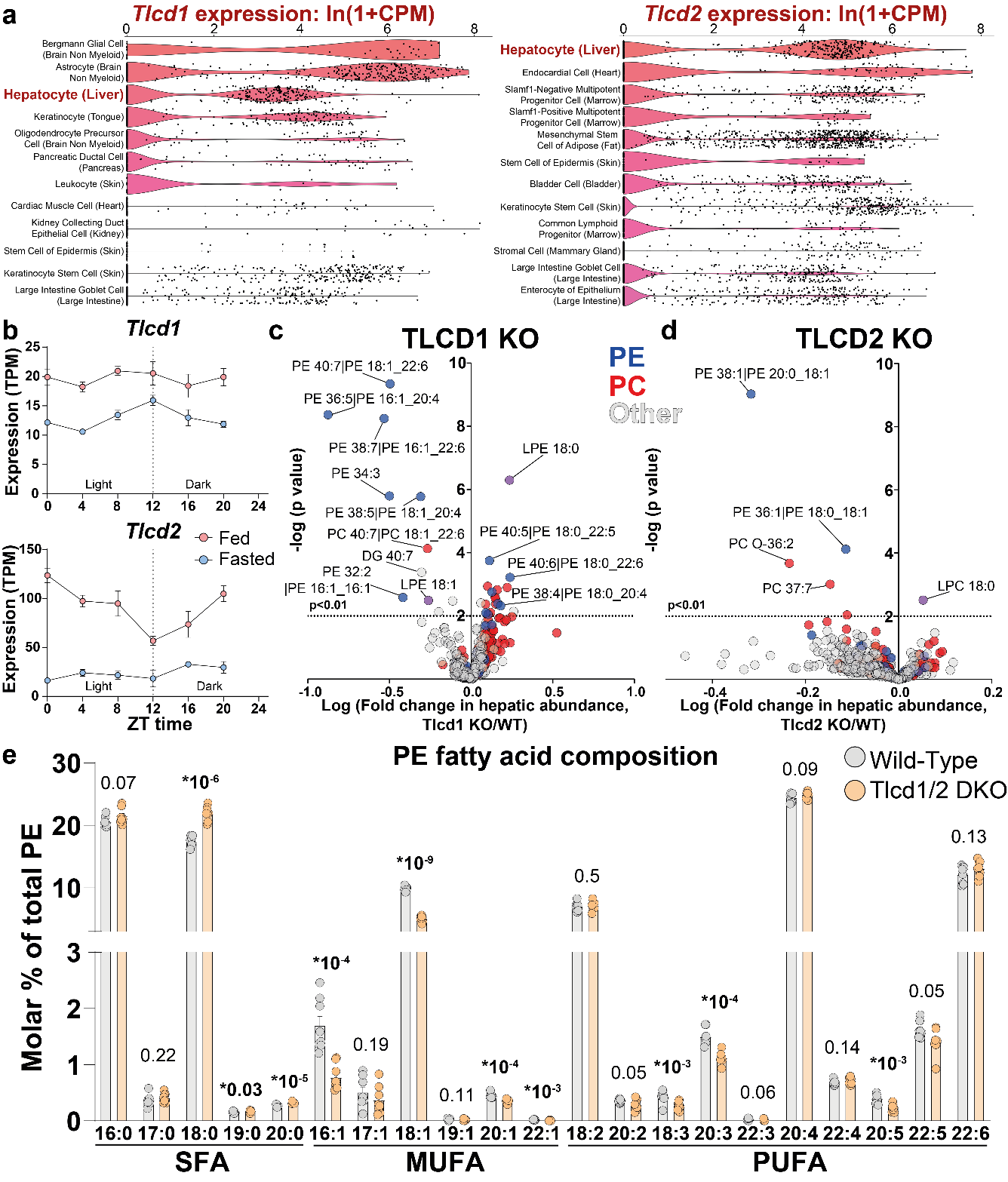


**Supplementary Fig. 1. Transcriptional regulation of *Tlcd1* and *Tlcd2* genes and their role in regulating mouse hepatic PE levels. a.** Violin plots of single-cell expression of *Tlcd1* and *Tlcd2* genes in the indicated mouse cell populations, ranked from highest to lowest average expression. Expression values are in ln(1+counts per million). Graphs were obtained and adapted from <https://tabula-muris.ds.czbiohub.org/>. **b.** *Tlcd1* and *Tlcd2* hepatic expression values in ad-libitum fed and 24 h-fasted mice at different times of day. N=3 mice/group/timepoint. Data obtained from Kinouchi *et al* (Ref. 18) and presented as mean values +/- SEM. **c.** Volcano plot of lipid species measured with high confidence (PC indicated in red and PE in blue) in wild-type and Tlcd1 KO, or **d.** wild-type and Tlcd2 KO chow-fed, 3-month-old male mouse livers (in **c**. n=5 Wild-Type and 10 Tlcd1 KO, in **d.** n=7 Wild-Type and 8 Tlcd2 KO). **e**. Complete fatty acyl chain profile of PE species measured in wild-type and Tlcd1/2 DKO chow-fed, 3-month-old male mouse livers (n=8 mice/group). In **d**-**e**, the logarithms of multiple unpaired two-tailed students t-test p values (not adjusted for multiple comparisons) are plotted on the y axis. In **e**, data are presented as mean values +/- SEM and two-tailed multiple t-test p values are indicated on graph. Source data for **b-e** are provided as a Source Data file.


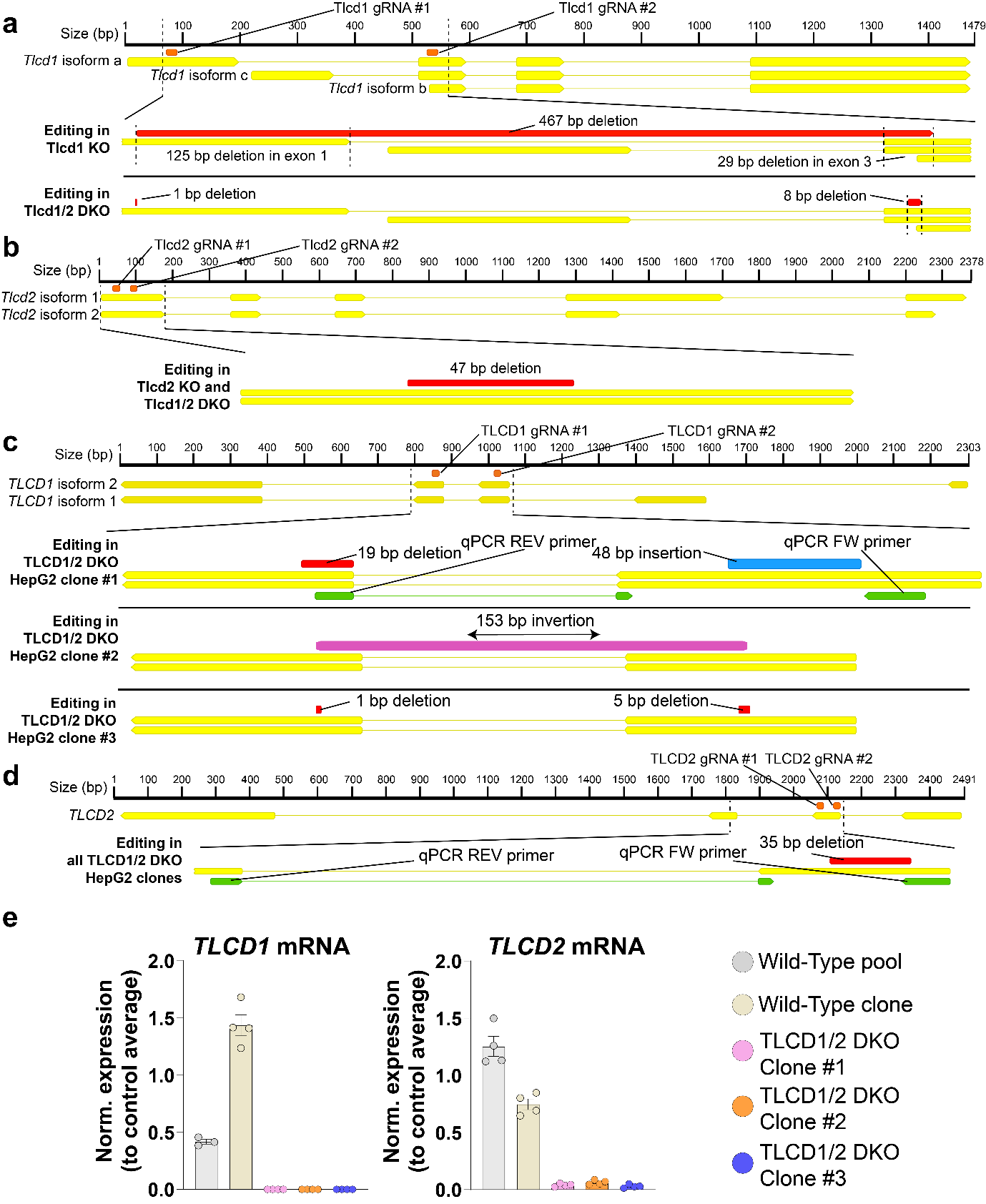


**Supplementary Fig. 2. Design and characterization of mouse and human *Tlcd1/2* genetic models. a.** CRISPR-edited mouse *Tlcd1* locus, **b.** mouse *Tlcd2* locus, **c.** human *TLCD1* locus, **d.** human *TLCD2* locus targeted with sgRNA pairs as indicated. SgRNA sequences and editing validation is described in Methods section. **e.** qPCR analysis of edited *TLCD1* and *TLCD2* mRNA, performed on a pool or single clone-derived HepG2 cells transfected with non-targeting gRNA, and on CRISPR-edited TLCD1/2 DKO three single clone-derived cell populations. Expression levels are normalized to the average of WT pool and WT clone averages, qPCR primer binding sites are indicated in **c** and **d**, n=4 technical replicates. In **e**, data are presented as mean values +/- SEM. Source data for **e** are provided as a Source Data file.


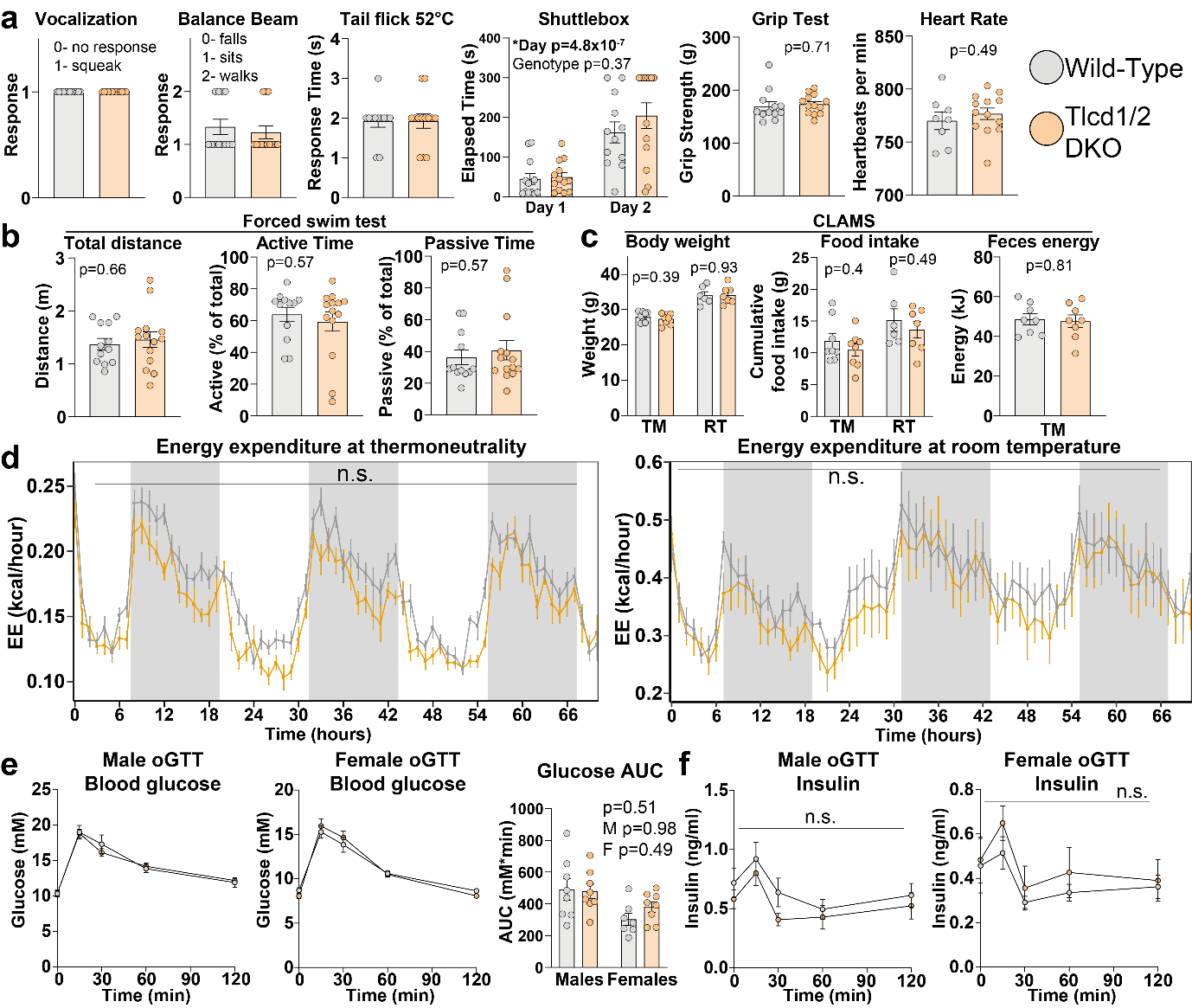


**Supplementary Fig. 3. Tlcd1/2 DKO mice have no developmental, behavioral, or metabolic phenotypes under standard housing conditions. a.** Indicated general phenotyping tests and **b.** performance during forced swim test of 3-5-month-old male wild-type (n=12) and Tlcd1/2 DKO (n=13) mice. **c.** Body weight for both thermoneutrality and room temperature groups; cumulative food intake and fecal energy for the thermoneutrality group; **d.** average energy expenditure plots during the CLAMS analysis of 5-month-old male wild-type and Tlcd1/2 DKO (n=8/group for TM, n=6/group for RT) mice. **e.** Blood glucose excursion curves and areas under curve, and **f.** circulating insulin during oral glucose tolerance tests of 5-month-old male (n=8/group) and female (n=7 wild-type, 8 DKO) mice. In **a-c**, two-tailed student’s t-test p values are indicated on graphs**.** For shuttlebox and oGTT AUC, p values are indicated on each graph for genotype factor in 2-way-ANOVA. All data are presented as mean values +/- SEM. Source data for **a-c** and **e-f** are provided as a Source Data file.


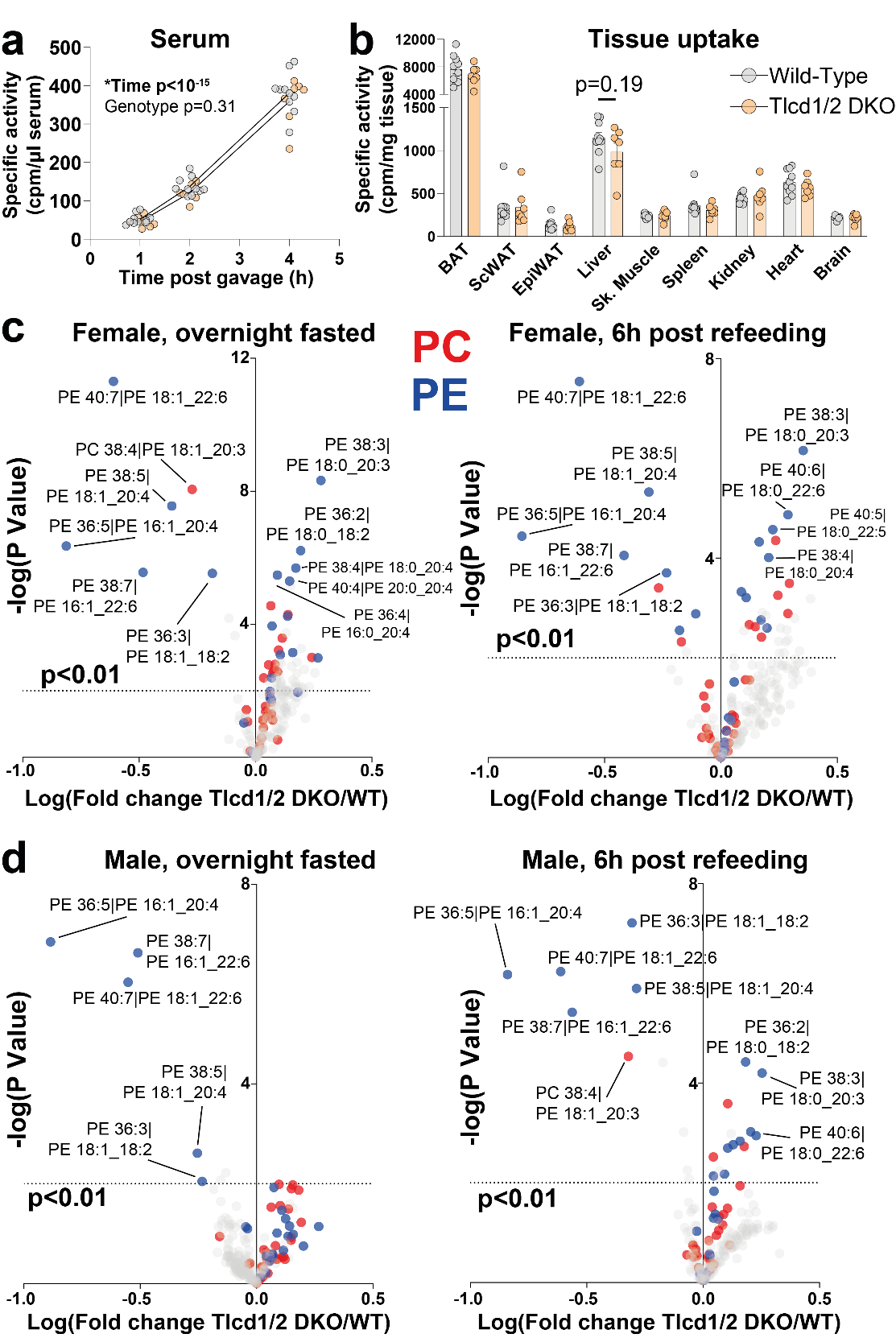


**Supplementary Fig. 4. TLCD1/2 function independently of cellular MUFA uptake or hepatic lipid metabolism. a.** Specific activity of serum sampled at indicated timepoints from 3-month-old female mice (n=10 wild-type, 7 Tlcd1/2 DKO) following oral administration of radiolabeled olive oil. **b.** Specific activity in the indicated tissue homogenates 4 h post oral gavage. **c.** Volcano plot of lipid species measured with high confidence (PC indicated in red and PE in blue) in wild-type and Tlcd1/2 DKO overnight fasted (n=6 wild-type, 6 DKO) or 6 h chow-refed (n=7 wild-type, 6 DKO) 3-month-old female, and **d.** overnight fasted (n=4 wild-type, 5 DKO) or 6 h chow-refed (n=4 wild-type, 5 DKO) 3-month-old male mouse livers. In **a**, p values are indicated on graph for time and genotype factors in repeated measures 2-way-ANOVA. In **b**, data are presented as mean values +/- SEM and two-tailed student’s t-test p value is indicated for liver and not shown for other tissues (all >0.2). In **c**-**d**, the logarithms of multiple unpaired two-tailed students t-test p values (not adjusted for multiple comparisons) are plotted on the y axis. Source data for **a-d** are provided as a Source Data file.


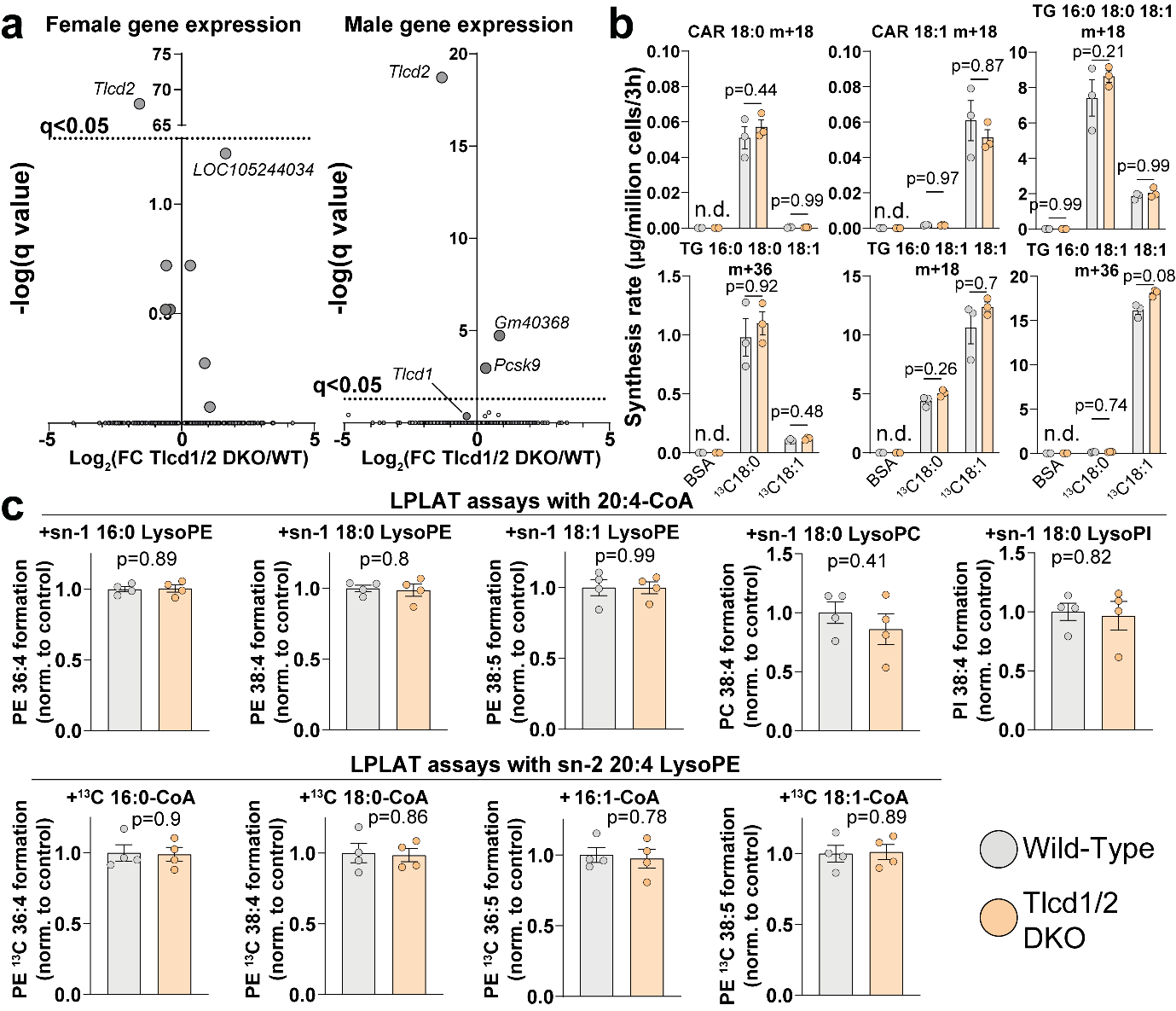


**Supplementary Fig. 5. TLCD1/2 proteins do not act by regulating gene expression or LPLAT enzymatic activity. a.** Volcano plots of hepatic gene expression in 10-month-old chow-fed female (n=10 WT, 9 Tlcd1/2 DKO) and male (n=9 WT, 7 DKO) mice. **b.** The levels of indicated acylcarnitine (CAR) and triglyceride (TG) species containing one (mass + 18 Da) or two (mass + 36 Da) stable labelled acyl chains in primary hepatocytes isolated from wild-type and Tlcd1/2 DKO chow-fed, 3-month-old female mice (n=3 mice/genotype), treated with 100 µM [U-^13^C]-18:0 or -18:1 for 3 h. **c.** The activity of indicated LPLAT reactions with microsomes isolated from chow-fed 3-month-old male wild-type and Tlcd1/2 DKO mouse livers (n=4 mice/group). In **b**, 2-way ANOVA Sidak’s multiple comparisons post-hoc test p values are indicated on graphs, and n.d. indicates undetectable levels of measured lipid. In **c**, two-tailed student’s t-test p values are indicated on graphs. In **b-c**, data are presented as mean values +/- SEM. Source data for **b-c** are provided as a Source Data file.


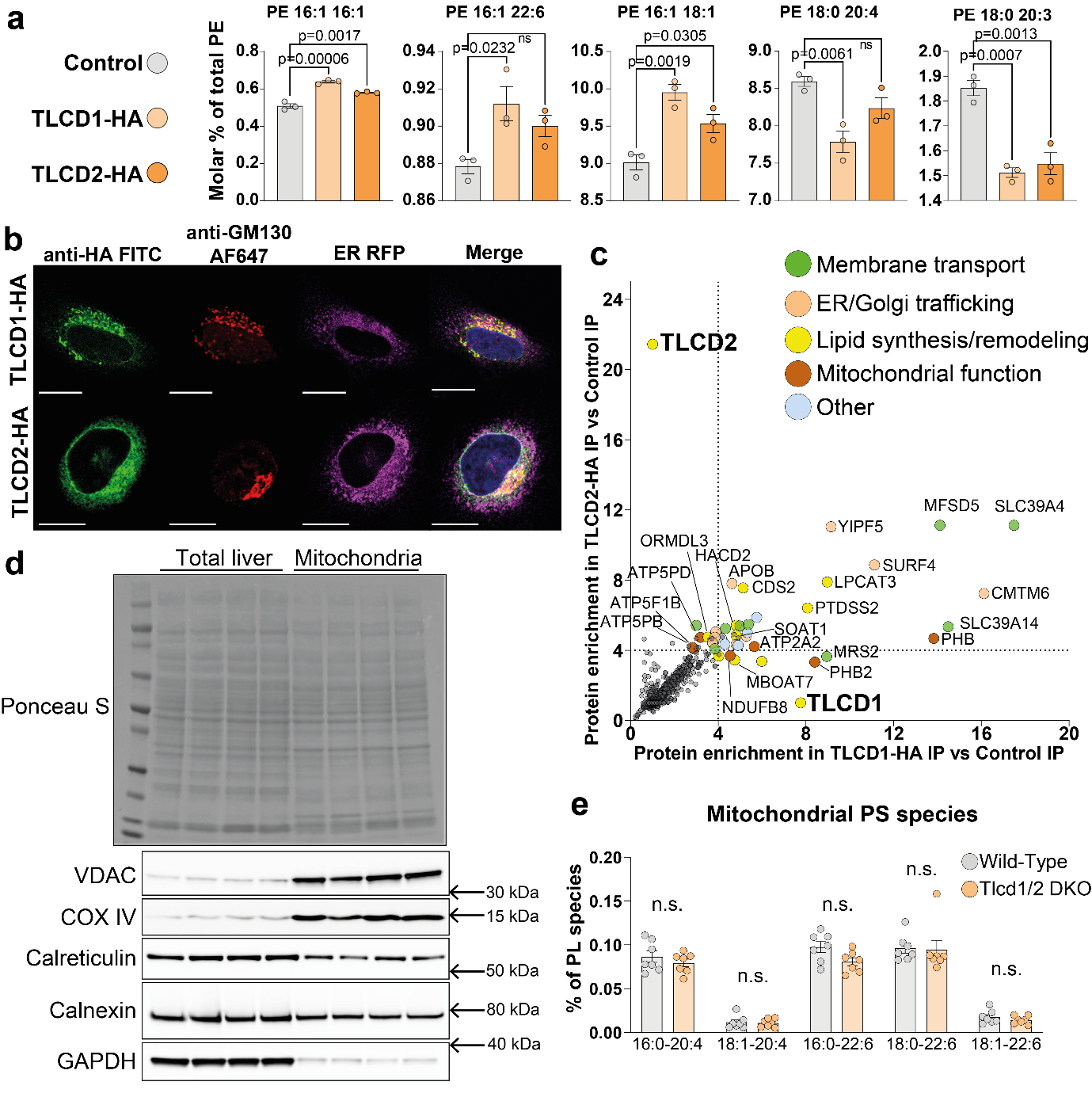


**Supplementary Fig. 6. Subcellular localization, protein interactome and the effects on the mitochondrial lipid composition by TLCD1/2 proteins. a.** The levels of indicated PE species measured in HepG2 cells with stable expression of HA-TLCD1 and HA-TLCD2 (n=3 technical replicates per condition). Note the Y axes do not start at 0 in the graphs. **b.** Representative (from 2 independent experiments, each containing >100 imaged individual cells) IHC images of HeLa cells with stable HA-TLCD1 or HA-TLCD2 expression stained as indicated above (GM-130 – Golgi marker). White scale bar = 20 µm. **c.** Proteins enriched in HA-TLCD1 IP compared to control plotted against those enriched in HA-TLCD2 IP compared to control. Proteins showing an arbitrary 4-fold enrichment in either IP are colored based on their biological function as indicated. **d.** Ponceau S total protein stain, and Western blots for VDAC and COX IV mitochondrial markers, Calreticulin and Calnexin ER markers, and GAPDH cytosol marker, of the proteins extracted from the total liver and isolated mitochondria from wild-type (n=2, lanes 1-2 and 5-6) and Tlcd1/2 DKO (n=2, lanes 3-4 and 7-8) chow-fed, 3-month-old male mice. Arrows on the right indicate the position of a nearest molecular weight marker for each blot. **e.** The levels of indicated phosphatidylserine (PS) species measured in the mitochondria isolated from the livers of wild-type and Tlcd1/2 DKO chow-fed, 3-month-old male mice (n=7 mice/genotype). In **a**, p values of 1-way-ANOVA with Sidak’s multiple comparisons test are indicated on graphs, n.s. – not significant. In **e**, n.s. indicates p>0.05 using two-tailed student’s t-test with Holm-Sidak’s correction for multiple testing. In **a** and **e**, data are presented as mean values +/- SEM. Source data for **a, d-e** are provided as a Source Data file.


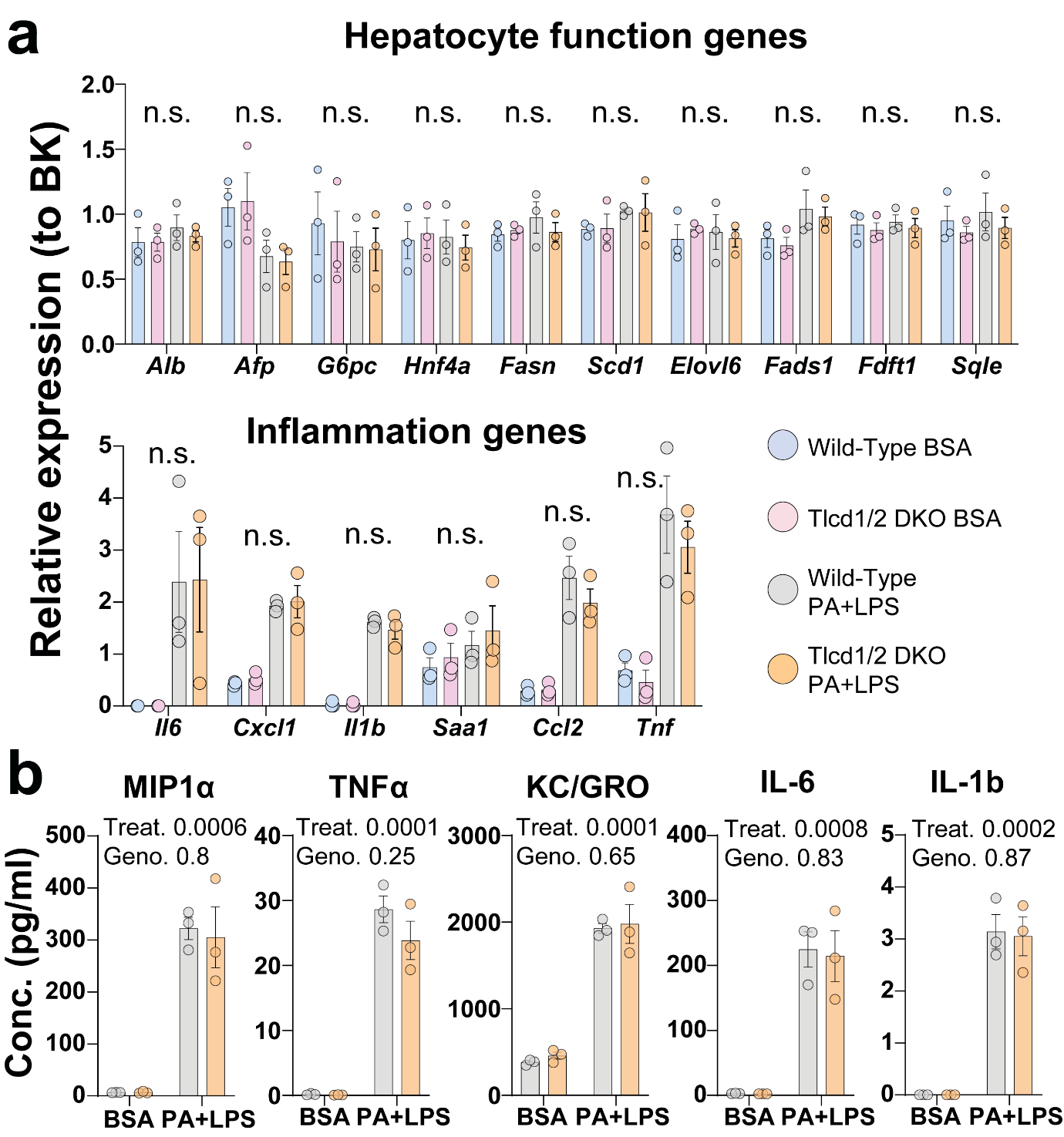


**Supplementary Fig. 7. The effects of *Tlcd1/2* deletion on the core gene expression and the inflammatory response of cultured mouse primary hepatocytes.** **a.** The expression of indicated lipogenic and hepatocyte marker genes, and **b.** cytokine secretion into culture supernatants of primary hepatocytes isolated from wild-type and Tlcd1/2 DKO chow-fed, 3-month-old female mice (n=3 mice/genotype), treated with a combination of 1 µM LPS and BSA-conjugated 250 µM PA, or BSA only control for 24 h. In **a**, n.s. indicates p>0.05 for genotype factor in 2-way-ANOVA. In **b**, p values for treatment and genotype factors in 2-way-ANOVA are indicated above graphs. All data are presented as mean values +/- SEM. Source data for **a-b** are provided as a Source Data file.


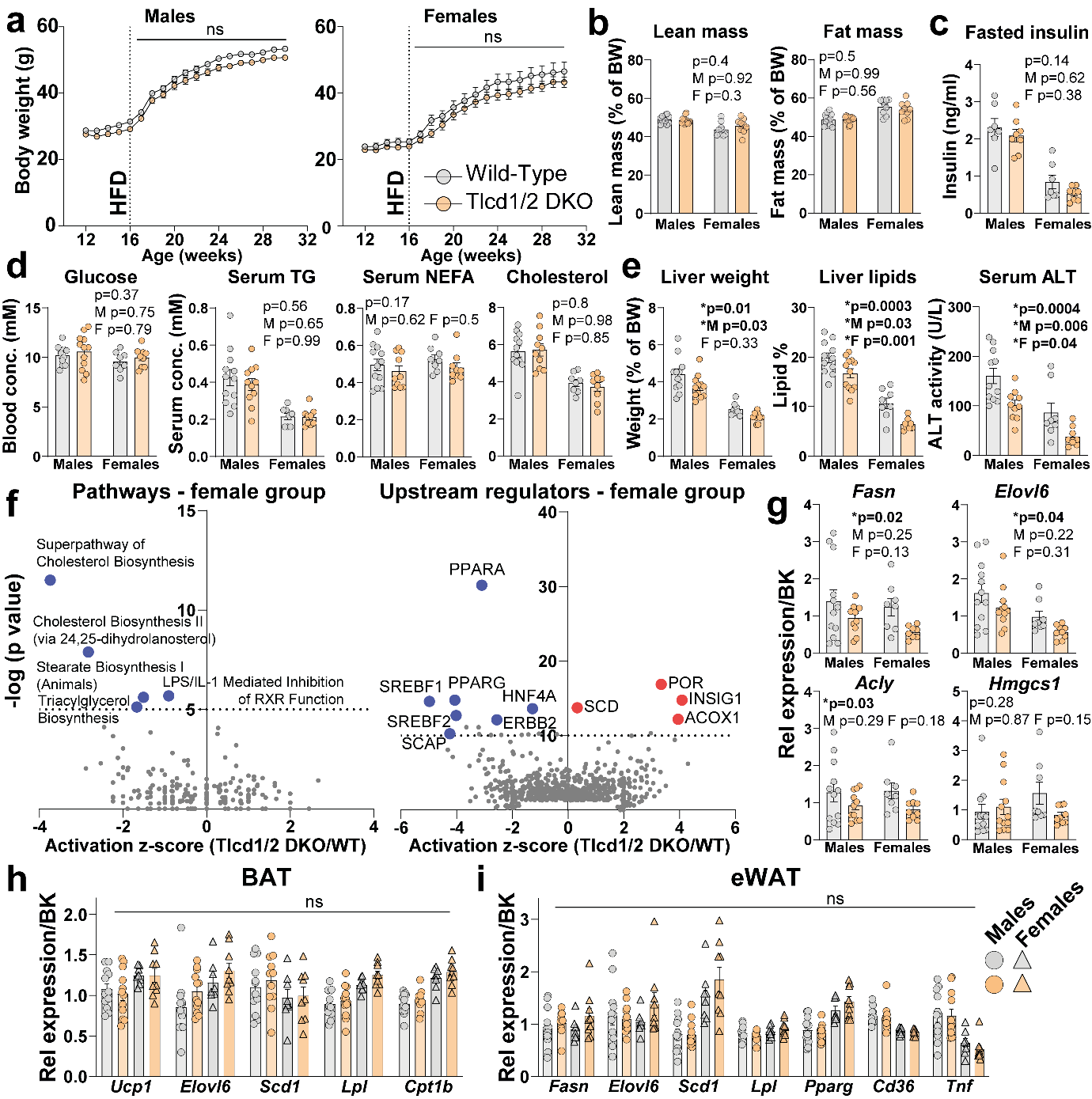


**Supplementary Fig. 8. Tlcd1/2 deficiency reduces liver lipid accumulation and serum ALT levels in HFD-fed mice. a.** Body weight curves, **b.** body composition measured by DEXA at 8 months of age, following 14 weeks of HFD, **c.** blood insulin levels measured in a subset of mice (n=7-8/group) after 6 hours fast. **d.** Blood glucose, circulating triglyceride, non-esterified fatty acid and cholesterol levels, **e.** Liver weights (normalized to body weights), liver lipid content and circulating ALT activity in 8-month-old HFD-fed wild-type and Tlcd1/2 DKO mice (n= 12 WT, 11 DKO males and 8 WT, 9 DKO females). **f.** Volcano plots of Ingenuity pathway and upstream regulator analyses of differentially expressed genes between HFD-fed Tlcd1/2 DKO (n=9) and wild-type (n=8) female groups. **g.** The expression of indicated genes in the livers, **h.** brown adipose tissues and **i.** epididymal white adipose tissues of HFD-fed wild-type and Tlcd1/2 DKO mice (n= 12 WT, 11 DKO males and 8 WT, 9 DKO females) In **a**, n.s. indicates p>0.05 between genotypes at each timepoint using repeated measures 2-way-ANOVA with Tukey’s multiple comparisons test. In **b**-**e**, **g**, p values are indicated on each graph for genotype factor in 2-way-ANOVA, with genotype differences in male and female groups evaluated using Sidak's multiple comparisons post-hoc test. In **f**, p values were calculated by Ingenuity Pathway Analysis software. In **h**-**i**, n.s. indicates p>0.05 for both overall genotype factor and male and female group comparison using 2-way-ANOVA with Sidak's multiple comparisons test, evaluated separately for each gene. All data are presented as mean values +/- SEM. Source data for **a-i** are provided as a Source Data file.


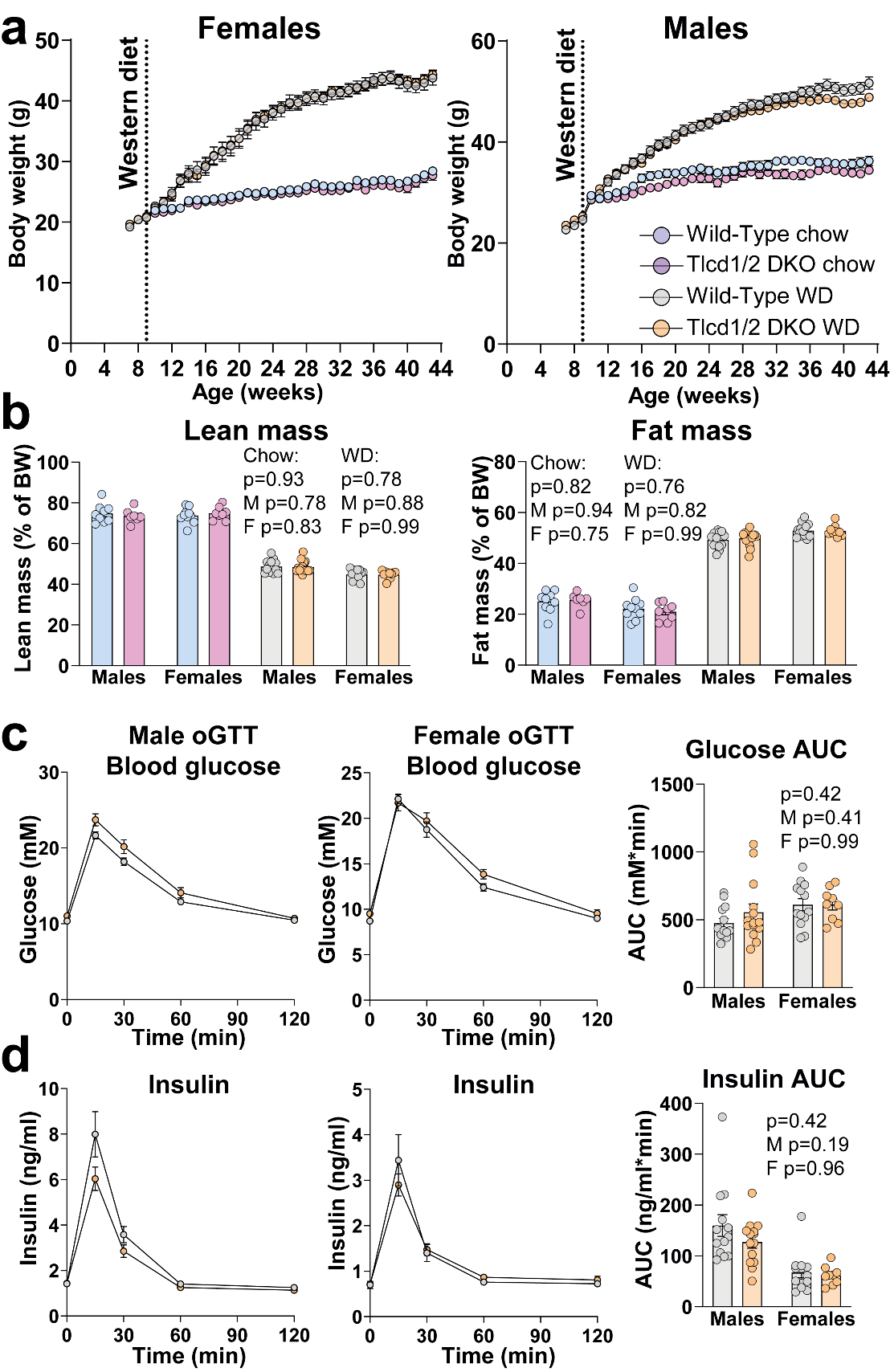


**Supplementary Fig. 9. WD-fed Tlcd1/2 DKO mice do not show differences in body weight gain, the acquisition of fat mass or the development of glucose intolerance compared to controls. a.** Body weight curves, **b.** body composition measured by DEXA at 9.5 months of age, **c.** blood glucose excursion curves and areas under curve, and **d.** circulating insulin and areas under curve during oral glucose tolerance tests performed at 9.75 months of age. N= chow-fed 9 WT, 7 DKO males and 10 WT, 9 DKO females, and WD-fed 13 WT, 14 DKO males and 13 WT, 9 DKO females. P values are indicated on each graph for genotype factor in 2-way-ANOVA, with genotype differences in male and female groups evaluated using Sidak's multiple comparisons post-hoc test. In **b**, statistical analyses on chow and WD groups were performed separately. All data are presented as mean values +/- SEM. Source data for **a-d** are provided as a Source Data file.


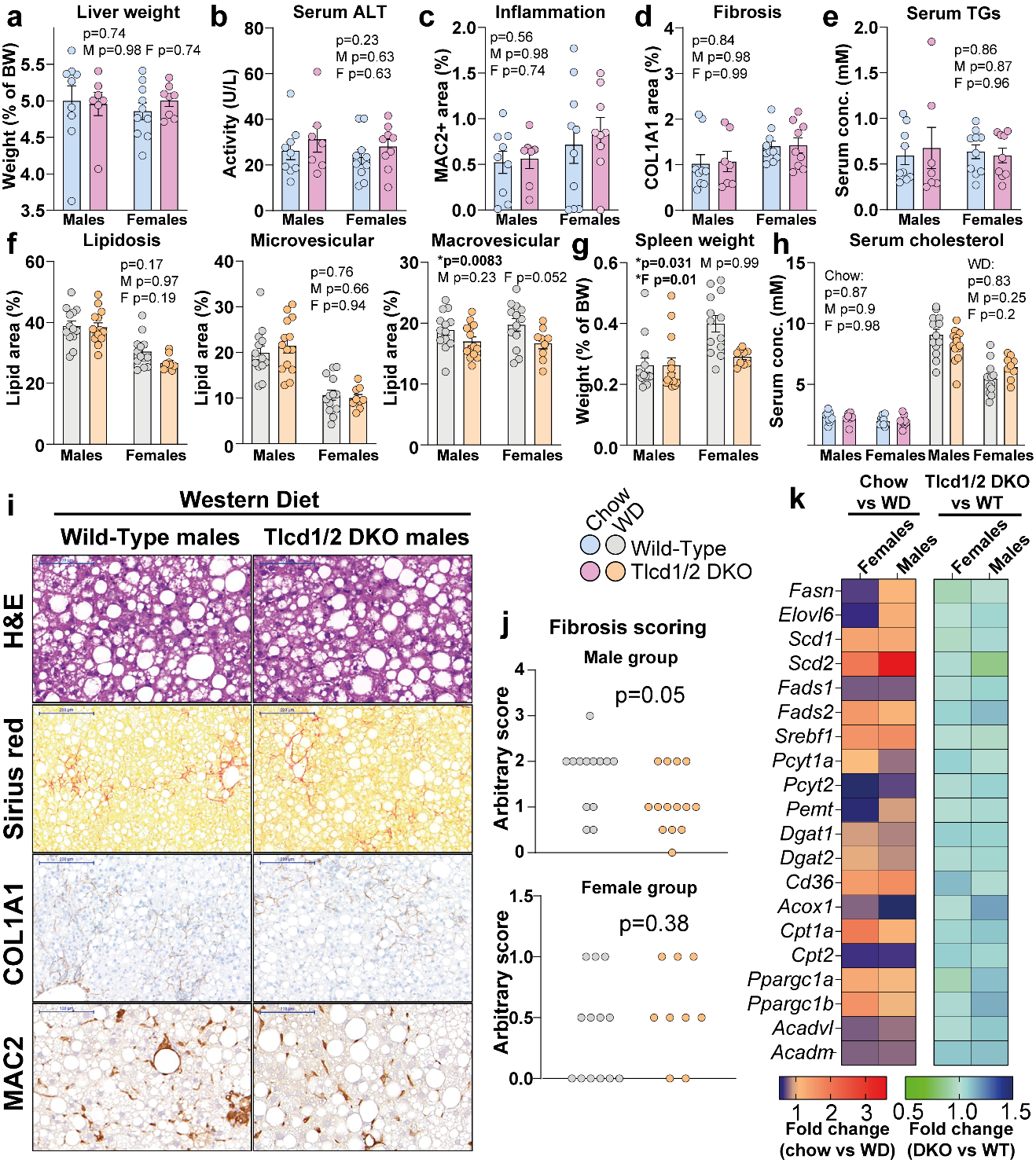


**Supplementary Fig. 10. Characterization of chow and WD-fed Tlcd1/2 DKO mouse liver phenotype. a.** Liver weights (normalized to body weights), **b.** circulating ALT activity, **c.** quantification of MAC2+ histological staining, **d.** quantification of COL1A1+ histological staining, **e.** circulating triglyceride levels, **f.** quantification of lipidosis in HE histological staining, **g.** spleen weights (normalized to body weights) and **h.** circulating cholesterol levels in 10-month-old chow- and WD-fed wild-type and Tlcd1/2 DKO mice (n= chow-fed 9 WT, 7 DKO males and 10 WT, 9 DKO females, and WD-fed 13 WT, 14 DKO males and 13 WT, 9 DKO females). **i.** Representative (from mice that were closest to the average value in each group) histological images of WD-fed male mouse livers. **j.** Qualitative fibrosis scoring of Sirius red staining evaluated by veterinary pathologist. **k.** The ratios between the average expression values of indicated hepatic genes in male and female groups. Left heatmap shows the ratios of WD/chow wild-type group average expression values, and the right heatmap – the ratios of Tlcd1/2 DKO/wild-type WD group average expression values. In **a-h**, P values are indicated on each graph for genotype factor in 2-way-ANOVA, with genotype differences in male and female groups evaluated using Sidak's multiple comparisons post-hoc test. In **j**, P values are indicated for each group evaluated using non-parametric two-sided Mann-Whitney ranks test. In **a**-**h**, data are presented as mean values +/- SEM. Source data for **a-h, j-k** are provided as a Source Data file.


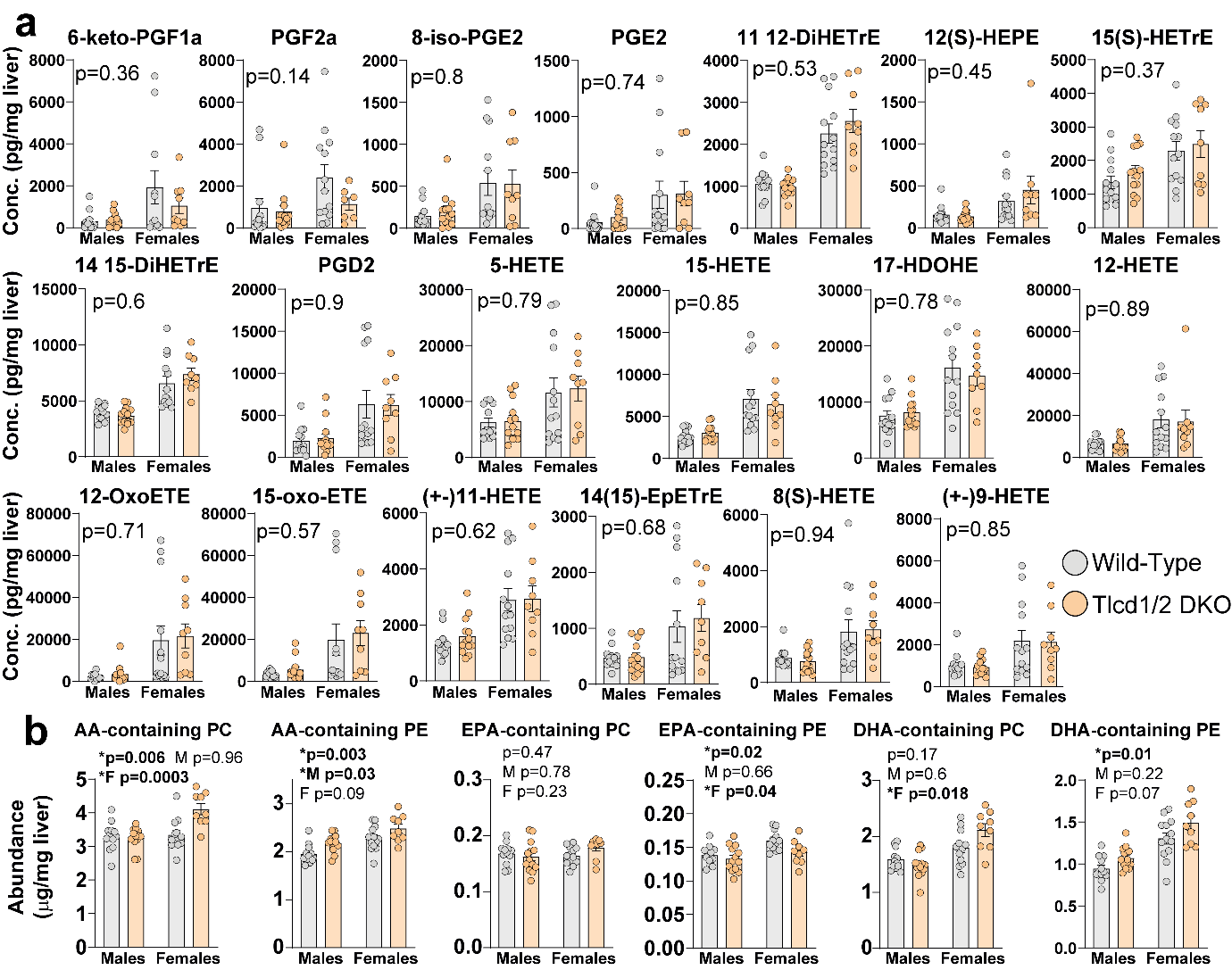


**Supplementary Fig. 11. Eicosanoid profiling and PUFA-containing PC and PE abundance in the livers of WD-fed Tlcd1/2 DKO mice. a.** The concentrations of indicated oxylipin species, and **b.** the abundance of AA (arachidonic acid, 20:4), EPA (eicosapentaenoic acid, 20:5) and DHA (docosahexaenoic acid, 22:6) containing PC and PE species, measured in the livers of WD-fed wild-type and Tlcd1/2 DKO mice (n= 13 WT, 13 DKO males and 13 WT, 9 DKO females). In **a.**, p values are indicated on each graph only for genotype factor in 2-way-ANOVA. In **a.**, all genotype comparisons in male and female groups have p>0.05 when evaluated using Sidak's multiple comparisons post-hoc test. In **b.**, p values are indicated on each graph for genotype factor in 2-way-ANOVA, with genotype differences in male and female groups evaluated using Sidak's multiple comparisons post-hoc test. All data are presented as mean values +/- SEM. Source data for **a** are provided as a Source Data file.
