# Supplementary material for: TLCD1 and TLCD2 regulate cellular phosphatidylethanolamine composition and promote the progression of non-alcoholic steatohepatitis
Source: Nat Commun. 2022 Oct 14;13:6020. doi: 10.1038/s41467-022-33735-6 (PMC9568529; doi:10.1038/s41467-022-33735-6)
Supplement: Supplementary file 2 — Reporting Summary [file 41467_2022_33735_MOESM2_ESM.pdf]

nature portfolio

Corresponding author(s): Kasparas Petkevicius

Last updated by author(s): Jul 29, 2022

Reporting Summary

Nature Portfolio wishes to improve the reproducibility of the work that we publish. This form provides structure for consistency and transparency in reporting. For further information on Nature Portfolio policies, see our Editorial Policies and the Editorial Policy Checklist.

Statistics

For all statistical analyses, confirm that the following items are present in the figure legend, table legend, main text, or Methods section.

n/a	Confirmed	
☐	☒	The exact sample size (*n*) for each experimental group/condition, given as a discrete number and unit of measurement
☐	☒	A statement on whether measurements were taken from distinct samples or whether the same sample was measured repeatedly
☐	☒	The statistical test(s) used AND whether they are one- or two-sided<br>*Only common tests should be described solely by name; describe more complex techniques in the Methods section.*
☐	☒	A description of all covariates tested
☐	☒	A description of any assumptions or corrections, such as tests of normality and adjustment for multiple comparisons
☐	☒	A full description of the statistical parameters including central tendency (e.g. means) or other basic estimates (e.g. regression coefficient) AND variation (e.g. standard deviation) or associated estimates of uncertainty (e.g. confidence intervals)
☐	☒	For null hypothesis testing, the test statistic (e.g. *F*, *t*, *r*) with confidence intervals, effect sizes, degrees of freedom and *P* value noted<br>*Give P values as exact values whenever suitable.*
☒	☐	For Bayesian analysis, information on the choice of priors and Markov chain Monte Carlo settings
☒	☐	For hierarchical and complex designs, identification of the appropriate level for tests and full reporting of outcomes
☒	☐	Estimates of effect sizes (e.g. Cohen's *d*, Pearson's *r*), indicating how they were calculated

Our web collection on statistics for biologists contains articles on many of the points above.

Software and code

Policy information about availability of computer code

Data collection	Integrated Oxymax software was used for mouse indirect calorimetry data acquisition (Columbus Instruments). Advanced Deep Scan AcquireX workflow (ThermoFisher) was used to acquire lipidomics data. ChemiDoc MP System (BioRad) software was used to generate Western blots.
Data analysis	Mouse indirect calorimetry data was analyzed using CalR software v1.3 (https://calrapp.org/). Visiopharm Integrator System software (v2019.2, Visiopharm) was used to analyze immunohistochemistry data. Cell immunofluorescence images were analyzed using ImageJ version 1.47 software. Lipidomics data was analyzed using MS-DIAL v4. Data from isotopically labeled samples was analyzed using a targeted method for lipids of interest using XCalibur Quan Browser (ThermoFisher). Proteomics data analysis was performed using Proteome Discoverer version 2.4 (ThermoFisher). RNA-seq and proteomics results were further analyzed using Ingenuity Pathway Analysis (QIAGEN).

For manuscripts utilizing custom algorithms or software that are central to the research but not yet described in published literature, software must be made available to editors and reviewers. We strongly encourage code deposition in a community repository (e.g. GitHub). See the Nature Portfolio guidelines for submitting code & software for further information.

Data

Policy information about availability of data

All manuscripts must include a data availability statement. This statement should provide the following information, where applicable:

- Accession codes, unique identifiers, or web links for publicly available datasets
- A description of any restrictions on data availability
- For clinical datasets or third party data, please ensure that the statement adheres to our policy

RNA-sequencing data have been deposited to Sequence Read Archive (SRA) database and are accessible through the SRA accession number PRJNA818121. Full lipidomics and proteomics datasets generated for this study are provided as Supplementary Datasets. All raw and unprocessed experimental data are available from

the corresponding author upon reasonable request.

Field-specific reporting

Please select the one below that is the best fit for your research. If you are not sure, read the appropriate sections before making your selection.

- [x] Life sciences
- [ ] Behavioural & social sciences
- [ ] Ecological, evolutionary & environmental sciences

For a reference copy of the document with all sections, see nature.com/documents/nr-reporting-summary-flat.pdf

Life sciences study design

All studies must disclose on these points even when the disclosure is negative.

Sample size	In all in vivo studies, sample sizes were determined using power calculations based on the variability of results from previous in-house in vivo studies. For long-term mouse dietary challenges, 2-3 additional mice were included in each group, to account for potential animal loss due to illness.<br>In the in vitro studies, n number was determined using power calculations based on our previous results and on published literature using the same cell models.
Data exclusions	In the NASH study, one Tlcd1/2 DKO mouse was removed from the male group (starting n=15, post exclusion n=14 that was presented in the figures) and two Tlcd1/2 DKO mice were removed from the female group (starting n=11, post exclusion n=9 that was presented in the figures). All exclusions were due to excessive scratch wounds in the neck area that could not be successfully treated.
Replication	Changes in hepatic PE composition were reproducibly measured by lipidomics in numerous Tlcd1/2 DKO mouse cohorts over a few generations - this is also evident in the data provided in the manuscript. Due to the complexity, time and cost of the experiments, high-fat diet and Western diet experiments were only performed once.<br>All cell culture and in vitro experiments were independently replicated at least twice, with all replication attempts showing similar results to what is presented in the paper.<br>Stable isotope fatty acid labeling primary hepatocyte experiment, and HepG2 immunoprecipitation-mass spectrometry experiments were performed once.
Randomization	For animal experiments, animal groups were determined based on the genotypes.<br>For cell experiments, groups were also determined by cell genotypes. In cell treatments, equal number of replicates from each genotype group were treated. Cells of different genotypes were seeded and treated in a way to evenly distribute different conditions across the edges and center of the plates.<br>In all sample analysis, all samples were randomized in the order they were analyzed. All lipidomics samples were processed using an automated robotic platform, thus ensuring same processing for all groups of samples.
Blinding	In all in vivo and mouse primary hepatocyte studies, investigators were blinded to the genotype of the animals, and could only access mouse IDs. Mouse IDs were matched to genotypes during data analysis step.<br>For all primary hepatocyte cell experiments, investigators were blinded to the genotype of each cell group and only marked each cell group with the donor mouse ID. The genotypes were revealed at the analysis step.<br>For cell line experiments, investigators were not blinded to group allocation, because the same investigator was involved in seeding the cells and treating them. As such, cell plates were labeled with the genotypes and the treatments.

Reporting for specific materials, systems and methods

We require information from authors about some types of materials, experimental systems and methods used in many studies. Here, indicate whether each material, system or method listed is relevant to your study. If you are not sure if a list item applies to your research, read the appropriate section before selecting a response.

Materials & experimental systems

n/a	Involved in the study	
[ ]	[x]	Antibodies
[ ]	[x]	Eukaryotic cell lines
[x]	[ ]	Palaeontology and archaeology
[ ]	[x]	Animals and other organisms
[x]	[ ]	Human research participants
[x]	[ ]	Clinical data
[x]	[ ]	Dual use research of concern

Methods

n/a	Involved in the study	
[x]	[ ]	ChIP-seq
[x]	[ ]	Flow cytometry
[x]	[ ]	MRI-based neuroimaging

Antibodies

Antibodies used	IHC: Anti-Mouse/Human Mac-2 (Galectin-3) monoclonal Rat Antibody (Clone M3/38), Catalog Number: CL8942AP, Company:

	CEDARLANE<br>IHC: COL1A1 / Collagen I Alpha 1 Rabbit anti-Mouse Polyclonal (aa1192-1207) Antibody, Catalog number: LS-C343921-100, Company: LSBio<br>IF: Recombinant Alexa Fluor® 647 Anti-GM130 Rabbit monoclonal antibody [EP892Y] - cis-Golgi Marker, Catalog number: ab195303, Company: Abcam<br>IF: FITC Anti-HA tag Rabbit polyclonal antibody, Catalog number: ab1208, Company: Abcam<br>WB: VDAC Rabbit Polyclonal Antibody, Catalog number: PA1-954A, Company: ThermoFisher<br>WB: COX IV Mouse monoclonal [mAbcam33985] Antibody, Catalog number: ab33985, Company: Abcam<br>WB: Calreticulin Rabbit monoclonal [EPR3924] Antibody, Catalog number: ab92516, Company: Abcam<br>WB: Calnexin Rabbit Polyclonal Antibody, Catalog number: PA5-34754, Company: ThermoFisher<br>WB: GAPDH Loading Control Monoclonal Antibody (GA1R), Catalog number: MA5-15738, Company: ThermoFisher<br>WB: Polyclonal Goat Anti-Rabbit Immunoglobulins/HRP secondary antibody, Catalog number: P0448, Company: Dako Agilent<br>WB: Polyclonal Goat Anti-Mouse Immunoglobulins/HRP secondary antibody, Catalog number: P0447, Company: Dako Agilent
Validation	VDAC, Calreticulin and Calnexin Western blot antibodies were validated by knockdown to ensure that the antibody binds to the antigen stated. The validation for these antibodies was performed by their commercial supplier and the validation figures are available on their website (ThermoFisher).<br>FITC Anti-HA tag immunofluorescence antibody was validated in-house. It did not give any signal in control cells but resulted in a fluorescence signal in cells expressing HA-tagged proteins.<br>The validation data for IHC antibodies are not available. However, both antibodies are against well established targets and are commonly used in the literature to assess liver inflammation and fibrosis.<br>The remaining Western blotting antibodies were not validated. However, they produced a clear single band on a Western blot at the expected molecular weight (can be seen in the Source Data provided), they were all against well-established protein markers and all antibodies were highly cited in the literature.

Eukaryotic cell lines

Policy information about cell lines

Cell line source(s)	HepG2 - ATCC, HeLa - ATCC
Authentication	HepG2 and HeLa lines were authenticated using STR fingerprint test and were 100% identity matched.
Mycoplasma contamination	HepG2 and HeLa lines were tested for Mycoplasma contamination by our CellBank team and were negative.
Commonly misidentified lines<br>(See ICLAC register)	No commonly misidentified lines were used.

Animals and other organisms

Policy information about studies involving animals; ARRIVE guidelines recommended for reporting animal research

Laboratory animals	All mice in this study were of C57Bl/6N genetic background. Tlcd1 KO, Tlcd2 KO and Tlcd1/2 DKO mice were generated as described in Methods. In most experiments, both male and female mice were used. The number of animals, their age and sex are clearly indicated in figure legends.<br>C57Bl/6N Tlcd1 KO and WT controls were both male and female at 3 months of age.<br>C57Bl/6N Tlcd2 KO and WT controls were both male and female at 3 months of age.<br>C57Bl/6N Tlcd1/2 DKO and WT control mice were both male and female. The youngest analyzed mice of this strain were 2 months of age (when their body weights were started to be measured). Most phenotyping tests and experiments on this strain were performed at 3-5 months of age. High fat diet experiment on this strain continued until 8 months of age. Western diet experiment on this strain continued until 10 month of age. The oldest analyzed mice of this strain were at 18 months of age.<br>C. elegans strains Pfld-1::FLD-1::GFP (from Ruiz et al, ref. 16) and pfat-7::GFP(rtIs30) were used. All worms were 3-day-old hermaphrodites.
Wild animals	No wild animals were used in this study.
Field-collected samples	No field-collected samples were generated or used in this study.
Ethics oversight	All mouse experiments were approved by the AstraZeneca internal committee for animal studies and the Gothenburg Ethics Committee for Experimental Animals compliant with EU directives on the protection of animals used for scientific purposes. The holding facility has received full accreditation from the Association for Assessment and Accreditation of Laboratory Animal Care (AAALAC).

Note that full information on the approval of the study protocol must also be provided in the manuscript.
